# Vitamin E in Plants: Biosynthesis Pathways, Biofortification Strategies, and Regulatory Dynamics

**DOI:** 10.3390/ijms26073380

**Published:** 2025-04-04

**Authors:** Yanjiao Li, Di Yang, Yuqing Ren, Yanzhong Luo, Hongyan Zheng, Yuan Liu, Lei Wang, Lan Zhang

**Affiliations:** 1 Biotechnology Research Institute, Chinese Academy of Agricultural Sciences, Beijing 100081, China; 82101212054@caas.cn (Y.L.); 82101222056@caas.cn (D.Y.); 82101232055@caas.cn (Y.R.); luoyanzhong@caas.cn (Y.L.); zhenghongyan@caas.cn (H.Z.); liuyuan03@caas.cn (Y.L.); 2 National Nanfan Research Institute (Sanya), Chinese Academy of Agricultural Sciences, Sanya 572024,China

**Keywords:** vitamin E, biosynthesis, biofortification, regulation

## Abstract

Vitamin E, mainly encompassing tocopherols and tocotrienols, is an essential antioxidant synthesized in the photosynthetic tissues of plants and photosynthetic bacteria, as well as in certain algae, yet dietary intake often falls short of recommended levels. Although synthetic supplements are available, natural vitamin E demonstrates higher bioavailability, creating a need for biofortification strategies to enrich crops with this nutrient. Recent advances in molecular genetics have elucidated key components of the vitamin E biosynthesis pathway, uncovering complex regulatory mechanisms and expanding opportunities for genetic enhancement. This review integrates current advances in vitamin E biosynthesis, novel gene discovery, diverse biofortification strategies, and insights into transporter-mediated regulation to enhance tocopherol and tocotrienol levels in staple crops. By aligning these advances, this review provides a framework to drive innovative biofortification efforts, positioning vitamin E enrichment as a sustainable solution for improved human and animal health.

## 1. Introduction

Vitamin E, initially identified in 1922 by Evans and Bishop as a growth factor essential for mouse fertility [1], has had its biosynthesis pathways and functional roles increasingly elucidated over time. It is primarily synthesized by photosynthetic organisms, such as plants and algae [2,3,4]. Vitamin E contributes significantly to cellular processes, including scavenging reactive oxygen species (ROS), preventing lipid peroxidation, and protecting photosystems. It also aids in stabilizing biological membranes, facilitating signal transduction and glucose transport, and bolstering plant resilience to abiotic stressors [5,6,7,8,9,10,11,12,13,14]. Vitamin E is a crucial micronutrient that humans and animals must obtain exogenously due to their inability to synthesize it. Beyond its nutritional value, vitamin E demonstrates diverse biological activities, including antioxidant effects, anti-tumorigenic properties, cholesterol-lowering actions, neuroprotection, and prevention of cardiovascular and cerebrovascular diseases [15,16,17,18,19,20,21,22,23,24]. In animals, vitamin E is essential for growth and development, often requiring substantial supplementation in animal feeds to meet nutritional needs [25,26].

Vitamin E, mainly comprising tocopherols and tocotrienols, is widely distributed in nature. Tocopherols are primarily found in plant leaves, dicot seeds, and monocot embryos, while tocotrienols are predominantly present in the endosperm of most monocots [27]. Despite its broad availability, low dietary intake of vitamin E remains a significant concern, with many populations experiencing chronic deficiencies [28,29,30]. Statistics up to 2024 show that 64% of the world’s population is deficient in vitamin E [31]. Observational studies suggest that a serum α-tocopherol concentration of over 30 μmol/L is associated with health benefits; however, a global survey indicated that only 21% of the population meets this threshold, highlighting widespread inadequacies in α-tocopherol levels [32].

In China, the “Reference Intake of Dietary Nutrients for Chinese Residents (2023 Edition)” recommends a daily intake of 14 mg α-tocopherol equivalents (α-TE) for its population [33]. The animal feed industry is also a major consumer of vitamin E, with China reportedly incorporating 17,500 tons of synthetic vitamin E into animal feed annually, resulting in an expenditure of approximately RMB 2.6 billion [25,26]. This rising demand highlights the significant economic impact of vitamin E supplementation.

Natural vitamin E, primarily occurring as d-α-tocopherol, exhibits higher bioavailability and potency than its synthetic counterpart, which consists mostly of dl-α-tocopherol-acetate, a mixture of stereoisomers in which only half align with the natural form, leaving the remainder poorly recognized by the human body [34,35]. Given vitamin E’s indispensable role in human and animal health, as well as the economic impacts of synthetic supplementation, boosting the vitamin E content of plants (particularly staple crops) has become a critical goal. Doing so would not only address nutritional deficiencies but also decrease dependency on synthetic supplements, providing a sustainable solution to meet the vitamin E needs of both humans and livestock.

Advances in science and technology have clarified the biosynthesis pathway of vitamin E, paving the way for genetic engineering approaches to enhance its levels in crops. Nonetheless, the varied outcomes of these biofortification strategies reveal the complexity of regulatory mechanisms within plant systems. Recently identified regulatory factors, including newly discovered genes in the vitamin E biosynthesis pathway, can synergistically improve biofortification when integrated with previously known target genes. Studies show that overexpressing these novel elements—alongside established genes—can produce larger increases in vitamin E content, thereby enhancing the effectiveness of biofortification strategies in a range of crops [36]. This review summarizes current knowledge of the vitamin E biosynthesis pathway and examines various biofortification strategies aimed at increasing vitamin E content in crops. Additionally, it investigates potential regulatory factors affecting vitamin E synthesis and explores the transport mechanisms of vitamin E and its precursors, proposing innovative strategies to strengthen biofortification efforts. Considering the varied metabolic pathways for tocopherols and tocotrienols across different plant species, the prospects for vitamin E biofortification remain highly promising. Targeted approaches, leveraging newly discovered regulatory factors and tailored to each crop’s specific metabolism, can significantly boost vitamin E levels and overall nutritional value. By synthesizing these insights, this review aims to guide future research and application on adopting distinct biofortification strategies for different crops.

## 2. The Synthetic Pathway of Vitamin E

**Figure 1:**
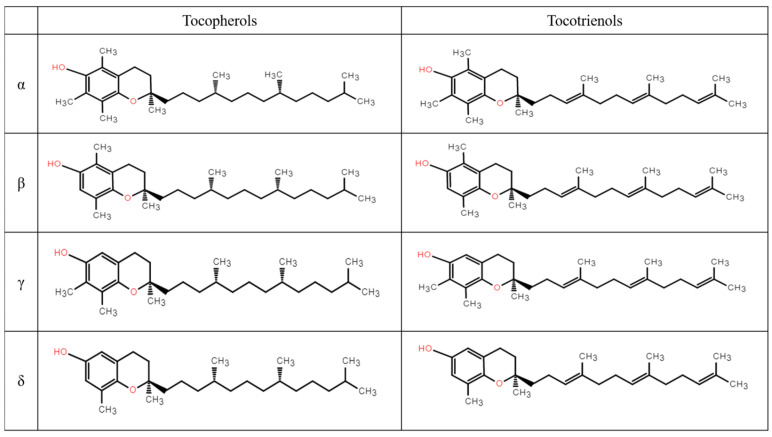
Chemical structures of the α-, β- γ-, and δ- homologs of tocopherols and tocotrienols.

The biosynthetic pathway of vitamin E, an amphipathic molecule distinguished by an aromatic ring head and a phytyl side chain tail, is intricately regulated within plastids [37]. Vitamin E mainly includes two primary groups, tocopherols and tocotrienols, which differ in the saturation of the phytyl side chain (Figure 1). Each group is further divided into four isomeric forms—α, β, γ, and δ—based on the number and position of methyl groups on the aromatic ring, with α-tocopherol displaying the highest biological activity [11,38].

**Figure 2:**
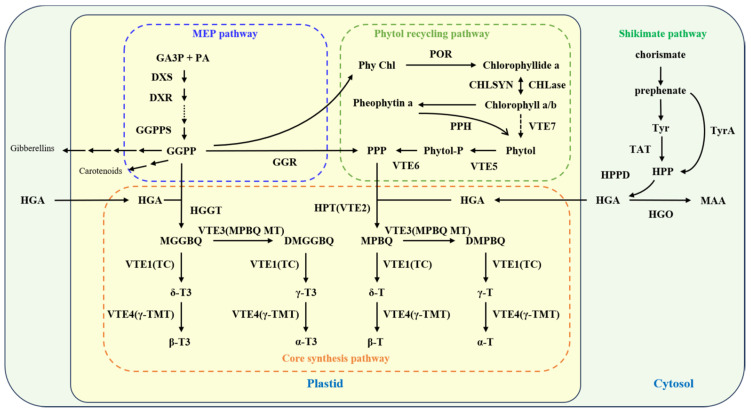
Vitamin E synthesis pathway. Abbreviations: MGGBQ: 2-methyl-6-geranylgeranylbenzoquinone; DMGGBQ: 2,3-methyl-5-geranylgeranylbenzoquinone; δ-T3: δ-tocotrienol; γ-T3: γ-tocotrienol; β-T3: β-tocotrienol; α-T3: α-tocotrienol; MPBQ: 2-methyl-6-phytylbenzoquinone; DMPBQ: 2,3-Methyl-5-phytylquinone; δ-T: δ-tocopherol; γ-T: γ-tocopherol; β-T: β-tocopherol; α-T: α-tocopherol; VTE3(MPBQ MT): 2-methyl-6-phytoquinone methyltransferase; VTE1(TC): tocopherol cyclase; VTE4(γ-TMT): γ-tocopherol methyltransferase; HPT(VTE2): homogentisate phytyltransferase; HGGT: homogentisate geranylgeranyl transferase; HGA: homogentisate; PPP: phytyl pyrophosphate; GGPP: geranylgeranyl pyrophosphate; GGR: GGPP reductase; CHLSYN: chlorophyll synthase; GG Chl: geranylgeranyl chlorophyll; phy Chl: phytyl chlorophyll; VTE5: phytol kinase; VTE6: phytyl monophosphate kinase; VTE7: hydrolase with α/β esterase activity; phytyl-P: phytyl monophosphate; GA3P: glyceraldehyde 3-phosphate; PA: pyruvate; MEP pathway: methylerythritol phosphate pathway; HPP: 4-hydroxypyruvate; HPPD: 4-hydroxypyruvate dioxygenase; Tyr: L-tyrosine; TAT: tyrosine aminotransferase; TyrA: prephenate dehydrogenase; MAA: 4-maleyl-acetoacetate, 4-Maleylacetoacetic acid; DXS: deoxyxylulose-5-phosphate synthase; DXR: deoxyxylulose-5-phosphate reductoisomerase; GGPPS: geranylgeranyl pyrophosphate synthase; CHLase: chlorophyllase; PPH: pheophytin hydrolase; HGO: HGA dioxygenase.

The core synthesis pathway of vitamin E begins with homogentisate (HGA) supplying the aromatic ring head, while geranylgeranyl-pyrophosphate (GGPP) and phytyl-pyrophosphate (PPP) contribute the phytyl side chains [39,40]. The enzyme homogentisate phytyltransferase (HPT/VTE2) catalyzes the condensation of HGA with PPP to form 2-methyl-6-phytylbenzoquinol (MPBQ), marking the commencement of tocopherol synthesis. Concurrently, homogentisate geranylgeranyltransferase (HGGT) facilitates the condensation of HGA with GGPP to form 2-methyl-6-geranylgeranylbenzoquinone (MGGBQ), initiating tocotrienol synthesis. Further enzymatic transformations convert MPBQ and MGGBQ into their respective derivatives, 2,3-dimethyl-5-phytylbenzoquinone (DMPBQ) and 2,3-dimethyl-5-geranylgeranylbenzoquinone (DMGGBQ), by MPBQ methyltransferase (MPBQ-MT, VTE3). Tocopherol cyclase (TC/VTE1) then catalyzes the conversion of these intermediates into δ-tocopherol, γ-tocopherol, δ-tocotrienol, and γ-tocotrienol. Subsequently, γ-tocopherol methyltransferase (γ-TMT/VTE4) methylates these compounds to produce β-tocopherol, α-tocopherol, β-tocotrienol, and α-tocotrienol (Figure 2).

Beyond the core pathway, the biosynthesis pathway of these precursors (HGA, PPP, GGPP) plays a crucial role in vitamin E synthesis. HGA is synthesized from 4-hydroxyphenylpyruvate (HPP) via HPP dioxygenase (HPPD) within the cytoplasmic shikimate pathway. In plants, HPP derives from tyrosine catabolism by tyrosine aminotransferase (TAT); in cyanobacteria, it is produced from chorismate/prephenate by chorismate/prephenate dehydrogenase (TyrA). The phytyl side chain precursors, GGPP and PPP, originate from the methylerythritol phosphate (MEP) pathway [27]. GGPP is synthesized by GGPP synthase (GGPPS) in plastids [41], and PPP is formed by the reduction of GGPP through GGPP reductase (GGR) [42]. PPP can also derive from chlorophyll biosynthesis and degradation, involving chlorophyll synthase (CHLSYN) [40,43], chlorophyllase (CHLase) [44], phytol kinase (VTE5), phytyl phosphate kinase (VTE6) [45,46,47], and hydrolase (VTE7) [36] (Figure 2). Because the biosynthesis of GGPP and PPP can vary widely among different plant species—both in terms of regulation and metabolic flux—biofortification strategies must be tailored to each species’ specific pathways. This targeted approach ensures that interventions effectively enhance vitamin E accumulation without disrupting other essential metabolic processes. In maize kernels, which have long been considered non-green tissues, research has identified two protochlorophyllide reductase (*POR*) genes associated with tocopherol content variations, confirming the roles of ZmPORB1 and ZmPORB2 in vitamin E synthesis [48,49,50]. One of the main challenges in manipulating regulatory genes such as *ZmPORB1* and *ZmPORB2* is achieving tissue-specific expression without disrupting essential metabolic pathways or compromising overall plant growth. Additionally, maintaining metabolic balance—so that resource allocation to these pathways does not negatively affect related processes—is crucial for sustaining healthy crop development. Depending on the vitamin E synthesis pathway, biofortification strategies may involve overexpressing a single gene, multiple genes, or directing gene expression to specific tissues or organs to optimize vitamin E content. For example, seed-specific promoters can be employed to drive *POR* expression while combining these regulators with other vitamin E biosynthetic genes. However, such approaches require comprehensive validation to ensure stable expression, minimal side effects, and robust performance under field conditions.

## 3. Genetic and Regulatory Analysis of Vitamin E Synthesis

It is clear that the biofortification of tocopherols remains challenging, while the biofortification of tocotrienols and the conversion between vitamin E components has proven more effective. It is conceivable that there may be many unknowns in the anabolic pathway of tocopherol. In recent years, researchers have studied the genetic characteristics of vitamin E synthesis through QTL, linkage mapping (LM), and genome-wide association studies (GWASs) in order to provide new insights for the anabolic metabolism and biofortification of vitamin E.

Researchers first analyzed the vitamin E synthesis pathway in Arabidopsis, involving 36 enzymes encoded by 53 genes [51,52], fully elucidating the synthesis pathway of vitamin E synthesis and the synthesis pathway of its precursors. In recent years, in-depth research has been conducted on maize, and association analysis and linkage analysis have been conducted on an association population consisting of 513 maize materials. It was found that there are nine single nucleotide polymorphism (SNP) sites near the *ZmTMT* gene that affect the α-tocopherol content; resequencing analysis found that InDel 7 located in the 5′ untranslated region (5′UTR) of the *ZmTMT* gene and InDel 118 located in the promoter region significantly affect the α-tocopherol content. Based on this, molecular markers were developed, which facilitated the molecular marker-assisted breeding of maize with high α-tocopherol content and provided a reference for other studies [53]. Through a GWAS analysis of the American maize NAM (nested association mapping) population, it was found that HGGT1 and γ-TMT in the vitamin E synthesis pathway were significantly correlated with vitamin E content [48]. These findings indicate that natural variation in vitamin E content is primarily governed by the *HGGT1* and *γ-TMT* genes, which explains the observed increase in tocotrienol levels through biofortification and the effective conversion between vitamin E components, while the impact of *HPT* overexpression remains moderate. This also suggests that vitamin E synthesis may be regulated by additional genes or genetic factors outside the core synthesis pathways. Genetic analysis in maize identified two SNP sites related to vitamin E synthesis within the intronic regions of a WRKY transcription factor gene and a *PPR* gene [53], which are hypothesized to influence vitamin E synthesis, though detailed research remains lacking. Diepenbrock et al. identified a gene within QTL39 encoding a zinc finger domain transcription factor, an eQTL influencing α-tocotrienol content [48]. In a GWAS analysis of the relationship between tocopherol and fatty acids in soybean populations, the transcription factor GmZF351, which regulates fatty acid synthesis, was found to directly activate genes in the tocopherol synthesis pathway (*Gmγ-TMT3*, *GmHPT*, *GmMPBQ-MT1*, and *GmHPPD1*), increasing fatty acid and tocopherol contents in soybean seeds. This provides evidence for regulatory factors that control the expression of key enzyme genes in the vitamin E synthesis pathway [54]. These studies collectively suggest that the synthesis of plant vitamin E is subject to complex regulatory control. However, research on the regulation of vitamin E synthesis remains limited, highlighting a promising area for future investigation.

## 4. Biofortification of Vitamin E

Given the significance of vitamin E and the elucidation of its biosynthesis pathway, considerable efforts have been made to undertake biofortification targeting the precursors and the key enzyme genes within this pathway. Broadly, two principal strategies are employed. Firstly, vitamin E levels could be augmented by adding vitamin E precursors directly to cell cultures, providing a rapid method to assess biofortification effectiveness. Secondly, overexpressing key enzyme genes within the vitamin E biosynthetic pathway in plants could modulate precursor substances such as HGA, PPP, and GGPP. This approach can be combined with the enhanced expression of genes encoding rate-limiting enzymes like HPT and HGGT, which catalyze initial steps in vitamin E synthesis. Alternatively, upregulating *γ-TMT* expression could adjust the vitamin E composition by attenuating the proportion of less active components and converting them into more biologically active forms. A key objective of this approach is to increase the concentration of α-tocopherol, the most active form of vitamin E. This can be accomplished, which facilitates the conversion of lower-activity tocopherols into α-tocopherol. These two strategies can be applied independently or in combination to optimize the biofortification process, aiming to enhance the nutritional quality and health-promoting properties of crops through advanced genetic and biotechnological interventions. Given the significance of vitamin E and the elucidation of its biosynthesis pathway, considerable efforts have been made to undertake the biofortification targeting of the key enzyme genes within this pathway. Broadly, two principal strategies are employed. The first strategy involves augmenting overall vitamin E levels by modulating precursor substances such as HGA, PPP, and GGPP, coupled with enhancing the expression of genes encoding rate-limiting enzymes like HPT and HGGT, which catalyze the initial steps in vitamin E synthesis. The second strategy involves modifying the vitamin E composition by attenuating the proportion of less active components and converting them into more biologically active forms. A key objective of this approach is to increase the concentration of α-tocopherol, the most active form of vitamin E. This can be accomplished by upregulating the expression of *γ-TMT*, which facilitates the conversion of lower-activity tocopherols into α-tocopherol. These two strategies can be applied independently or in combination to optimize the biofortification process, aiming to enhance the nutritional quality and health-promoting properties of crops through advanced genetic and biotechnological interventions.

### 4.1. Addition of Vitamin E Precursors to Cell Cultures

Exogenous supplementation in plant cell cultures is a foundational approach to examining its effects on metabolite levels, particularly in vitamin E synthesis [55]. Studies reveal that the impact of added precursors varies with their type and concentration. In sunflower cell cultures, HGA supplementation at concentrations of as low as 100 mg/L and as high as 200 mg/L led to a 1.3-fold increase in α-tocopherol content, whereas the supplementation with phytol had no significant effect on tocopherol accumulation [56]. In safflower cultures, the tocopherol levels remained unchanged 3 days after the addition of HGA, but phytol addition led to a notable increase. After 14 days, both HGA and phytol supplementation led to significant enhancements in tocopherol content, showing a 3.3-fold and 18.4-fold increase, respectively [57]. In soybean suspension cultures, the tocopherol content tripled with the individual addition of HGA or phytol. When both were added together, the tocopherol content surged to six times the control level [58]. In some plant species, the efficacy of exogenous HGA and phytol supplementation is significantly influenced by species-specific differences in metabolic flux, subcellular compartmentalization, and regulatory control of key biosynthetic enzymes, causing variable responses to these precursors. Additionally, variations in enzyme expression, transport systems, and interactions with parallel pathways—such as chlorophyll biosynthesis, which also relies on phytol—can affect how efficiently supplemented HGA and phytol are converted into tocopherols. Consequently, future research should focus on identifying and alleviating metabolic bottlenecks, refining supplementation protocols (e.g., optimal dosages, delivery methods, timing), and exploring synergistic strategies (like combining supplementation with the overexpression of critical biosynthetic genes) to maximize vitamin E accumulation while sustaining normal plant growth.

Further, these cell cultures allow researchers to maintain precise control overgrowth conditions, nutrient supply, and precursor concentrations, making it easier to evaluate changes in vitamin E accumulation. Compared to in vivo approaches with whole plants, cell culture methods are faster and more amenable to large-scale screening; however, they do not fully replicate the complexity of intact organisms, where tissue-specific transport, developmental stages, and environmental factors can significantly influence vitamin E metabolism. Consequently, while in vitro experiments offer valuable insights and a foundation for optimizing precursor supplementation, subsequent validation in whole plants—ideally under field conditions—is essential to confirm real-world applicability.

### 4.2. Overexpression of Genes Involved in Vitamin E Biosynthetic Pathway in Plants

#### 4.2.1. Boosting the Synthesis of HGA

The overexpression of *HPPD*, a crucial enzyme in the synthesis of HGA, has yielded varying results across different organisms in terms of vitamin E enhancement. In transgenic Arabidopsis, tobacco, and soybean, *HPPD* overexpression did not significantly increase the vitamin E content [58,59,60,61,62]. In contrast, in cyanobacteria, HPPD overexpression resulted in a 7-fold increase in tocopherol content [58]. This disparity is likely due to differences in the HPP biosynthetic pathways between plants and bacteria. In bacteria, TyrA directly converts prephenate to HPP, whereas plants follow a more complex pathway, with tyrosine as an intermediate that feedback inhibits TyrA, tightly regulating HPP production and consequently limiting HGA and vitamin E synthesis [63]. Further research showed that the co-overexpression of *HPPD* and *TyrA* in Arabidopsis and tobacco significantly increased the vitamin E content, suggesting that enhancing HGA synthesis could boost vitamin E levels, particularly tocotrienols, in plants [60,62]. In transgenic Arabidopsis, canola, and soybean, this genetic modification resulted in notable increases in vitamin E content, highlighting the pivotal role of HGA in tocotrienol synthesis [58]. These findings suggest that while HGA availability is crucial for tocotrienol synthesis, its influence on tocopherol production may be less pronounced, reflecting a nuanced regulatory mechanism in vitamin E biosynthesis in plants. The simultaneous overexpression of *HPPD* and *TyrA* genes in transgenic Arabidopsis and soybean seeds led to substantial increases in HGA content—60-fold and 800-fold, respectively—resulting in the noticeable darkening of seed color [58]. However, this increase did not correlate with a proportional rise in vitamin E levels, suggesting that only a fraction of the newly synthesized HGA contributes to vitamin E synthesis, with possible alternative metabolic pathways for HGA utilization. In the soybean MO12 mutant, the mutation of HGA dioxygenase 1 (HGO1) caused a 30-fold increase in HGA but only a twofold increase in vitamin E content, indicating a regulatory mechanism that restricts HGA use for vitamin E synthesis [64]. In camelina, the simultaneous overexpression of *HPPD* and *TyrA* genes led to a 2.5-fold increase in vitamin E content, while the RNAi inhibition of *HGO* expression raised the vitamin E content by about 1.4 times. However, when *HGO* expression was inhibited in the transgenic lines overexpressing *HPPD* and *TyrA*, no further increase in vitamin E was observed [65]. HGO1’s role in converting HGA to MAA highlights its potential as a bottleneck in vitamin E biosynthesis. Notably, as in transgenic plants with elevated HGA, the rise in vitamin E levels in the MO12 mutant was primarily attributed to an increase in tocotrienols, without a significant change in tocopherol levels [64]. This reinforces the notion that HGA availability alone does not determine tocopherol levels. This observation raises a pertinent question regarding the role of other precursors like PPP or GGPP in vitamin E synthesis. Are these compounds more critical in determining the production of vitamin E, particularly tocopherols? Further investigation into the roles of PPP and GGPP may elucidate their relative importance to HGA in vitamin E biosynthesis and potentially reveal new biofortification targets to enhance tocopherol content in plants.

#### 4.2.2. Genetic Enhancement of GGPP and PPP Contents

GGPP is a crucial precursor for several plant metabolites, including vitamin E, carotenoids, chlorophyll, gibberellins, and diterpenes, positioning GGPP synthesis as a key juncture in isoprenoid biosynthesis. Enhancing the expression of enzymes upstream in the GGPP synthesis pathway has been employed as a strategy to increase GGPP availability. The overexpression of deoxyxylulose-5-phosphate synthase (DXS), the initial rate-limiting enzyme in the MEP pathway, led to an approximate 2-fold increase in tocopherol content in Arabidopsis seedlings, primarily in the form of α-tocopherol [66]. This suggests that elevated DXS expression raises GGPP levels, subsequently increasing PPP and tocopherol production. However, this enhancement in isoprenoid content was not observed in mature plant leaves, indicating that DXS’s effect may be limited to younger tissues [67]. In contrast, overexpressing deoxyxylulose-5-phosphate reductoisomerase (DXR), the second key enzyme in the MEP pathway, did not result in a similar increase in tocopherol content, suggesting potential regulatory mechanisms or substrate limitations affecting pathway flux. Specifically, the concentration of D-5-deoxyxylulose phosphate, a substrate in the MEP pathway, may limit the effectiveness of *DXR* overexpression in boosting isoprenoid production [68]. To address the limitations in DXP production when overexpressing *DXR*, one potential strategy is co-expressing upstream genes like *DXS* (which synthesizes DXP) alongside *DXR* to ensure a balanced flux through the MEP pathway. Additionally, investigating other rate-limiting enzymes and regulatory elements in the MEP pathway (such as GcpE, LytB, or transcription factors that coordinate chloroplast development) could offer further avenues to enhance isoprenoid production—particularly in mature tissues, where physiological constraints often reduce metabolic plasticity. Similarly, increasing the expression of key enzymes in the PPP synthesis pathway has been used to improve the effectiveness of PPP. Studies in rice have shown that products of both GGR1 and GGR2 exhibit GGR activity, with double mutations in these genes significantly reducing tocopherol content, underscoring GGR’s critical role in tocopherol synthesis [69]. Additionally, in maize, the overexpression of *ZmPORB* increased the tocopherol content by 1.5-fold in leaves and 1.19-fold in grains [50]. In Arabidopsis, the *vte7* mutant showed a 0.45-fold reduction in seed tocopherol content without a decrease in leaves, while in maize, the *ZmVTE7* mutation reduced the total vitamin E content by 0.62-fold in kernels and 0.51-fold in leaves [36]. The overexpression of *AtVTE7* and *ZmVTE7* partially restored seed tocopherol levels and significantly increased the leaf tocopherol content by 3.6-fold and 6.9-fold in *Atvte7* [36]. In Arabidopsis, the *vte5* mutant showed a reduction in tocopherol to 0.2-fold in seeds and 0.35-fold in leaves, which was almost recovered by *VTE5* overexpression. Additionally, *VTE5* overexpression in wild-type plants slightly increased tocopherol levels [70]. Transgenic Arabidopsis seeds expressing the *VTE6* gene showed a 1.15-fold increase in tocopherol content [47]. In green seeds, the simultaneous inhibition of *CHLSYN* and overexpression of *HPT* increased the tocopherol content in Arabidopsis seeds by over 2-fold [43]. These findings indicate that boosting PPP and GGPP levels has a more limited effect on overall vitamin E enhancement but notably influences the tocopherol content in green seeds. This suggests that other regulatory factors or mechanisms may influence tocopherol biosynthesis efficiency in plants. Collectively, these findings highlight the potential to amplify vitamin E accumulation by strategically combining these genes in plants.

#### 4.2.3. Overexpression of Rate-Limiting Enzymes HPT and HGGT

The enzymes HPT and HGGT play crucial roles in vitamin E biosynthesis, rendering them key targets for genetic engineering aimed at enhancing the total vitamin E content. The overexpression of *AtHPT* in various plants has led to significant increases in α-tocopherol levels across different tissues, although the extent varies by plant species and tissue type. For example, the overexpression of *AtHPT* increased α-tocopherol levels in the leaves of Arabidopsis, tobacco, potato, lettuce, and rice by 4.2, 5.4, 4.6, 2.7, and 5.3 times, respectively [36,58,71,72,73,74,75]. However, these studies also showed that tocopherol increases in other organs were less pronounced, particularly in soybean, rapeseed, and potato tubers, where levels increased by only 1.75, 1.2, and 2.2 times, respectively. This suggests that *HPT* overexpression may have a more substantial impact on tocopherol biosynthesis in vegetative tissues compared to reproductive tissues, such as seeds. Further experiments with *HPT* overexpression from different sources, such as apple *MdHPT1* in tomato and lettuce in Arabidopsis, also led to substantial increases in α-tocopherol content in leaves by 3.6 and 18 times, respectively [76,77]. Combining the overexpression of *HPT* with other genes in the vitamin E pathway has shown additive effects, particularly in leaf tissues, though less so in seeds. For instance, the co-overexpression of *AtHPT* with *AtVTE7* in Arabidopsis [36] and with *AtTC* in rice [74] led to substantial increases in leaf tocopherol levels by 7.4 and 5.3 times, respectively. These findings suggest that while HPT activity is crucial for tocopherol synthesis in leaves, it may not be the primary limiting factor in seed tocopherol biosynthesis. This indicates a nuanced regulation of tocopherol biosynthesis across different plant tissues and developmental stages, providing valuable insights for targeted biofortification strategies to enhance the vitamin E content. The overexpression of the barley *HvHGGT* has been shown to increase tocotrienol levels across various crops, approximately 1.1 to 1.15-fold in barley grains, 6-fold in maize kernels, 10~15-fold in Arabidopsis leaves, and 10-fold and 3-fold in soybean and upland cotton grains, all with notable increases in the tocotrienol content [7,78,79,80]. Moreover, overexpressing rice *OsHGGT* in tobacco resulted in a 3.4~4.7-fold increase in leaf vitamin E content [81]. This indicates that HGGT is pivotal in tocotrienol biosynthesis. However, in maize, while tocotrienol levels in kernels and extracted oil increased 18-fold, tocopherol content decreased by 18% [82], suggesting a potential metabolic trade-off between tocopherol and tocotrienol biosynthesis when *HGGT* is overexpressed. These findings imply that while HGA, a precursor for both tocopherols and tocotrienols, is vital, its availability may limit synthesis under high demand, affecting the balance between tocopherol and tocotrienol accumulation. Thus, enhancing HGGT activity is a promising strategy for boosting tocotrienol levels, but the impact on overall vitamin E composition and potential trade-offs with tocopherol synthesis must be carefully considered in biofortification efforts. Overall, there have been no reports of adverse effects of the overexpression of *HPT* and *HGGT* on plants.

#### 4.2.4. Increase the Content of Highly Active α-Tocopherol

The overexpression of *γ-TMT* boosts the conversion of less active vitamin E forms (γ- and δ-tocopherol/tocotrienol) into their more potent α- and β-counterparts, yet research has primarily centered on α-tocopherol because (1) α-tocopherol is preferentially recognized by the α-tocopherol transfer protein (α-TTP) in humans and animals, and (2) the δ- and β- forms typically occur at lower levels and exhibit lower bioactivity. Consequently, enhancing α-tocopherol emerges as a key focus in biofortification efforts. The overexpression of the *γ-TMT* gene has been shown to significantly increase the α-tocopherol content by 80-fold and shift the α-/γ-tocopherol ratio from 0.01 to 13 across various plant species, underscoring its potential in biofortification strategies to improve the vitamin E profile in crops, indicating the naturally low activity of γ-TMT in wild-type seeds [83]. Similar enhancements were observed in transgenic lettuce T2 lines expressing the Arabidopsis *AtTMT* gene, where the α-/γ-tocopherol ratio increased from 1.0 to 138.0 [84]. Further, the overexpression of *AtTMT* in mustard and tobacco resulted in 6-fold and 9.6-fold increases in α-tocopherol content, respectively [85,86]. In soybeans, the introduction of *AtTMT*, *PfTMT* (from *Perilla frutescens*), and *BnTMT* (from *Brassica napus*) increased the α-tocopherol content in seeds by 4, 10.4, and 11.1-fold, respectively, illustrating the efficacy of cross-species gene transfers in enhancing α-tocopherol levels [87,88,89]. Similarly, the overexpression of *MsTMT* in Arabidopsis demonstrated an 11-fold increase in seed α-tocopherol content [90,91]. The co-overexpression of AtTMT and AtHPT in potato led to a 2.6 to 2.8-fold increase in α-tocopherol content in tubers and a 4.8 to 5.1-fold increase in leaves, suggesting a synergistic effect [75]. In maize, the overexpression of soybean *GmTMT2a* and maize *ZmTMT* converted over 90% of γ-tocopherol in kernels to α-tocopherol [92,93], significantly altering the vitamin E profile and also increasing the α-tocotrienol content [94]. Overexpressing the *γ-TMT* gene specifically enhances levels of α-tocopherol, the most biologically active form of vitamin E, without affecting the total tocopherol content. This targeted increase highlights γ-TMT’s role in improving plant nutritional quality by selectively enriching the α-form content while maintaining overall vitamin E quantity. Similarly, the overexpression of the *MPBQ MT* does not modify the total tocopherol content but alters the tocopherol composition. The concurrent overexpression of *γ-TMT* and *MPBQ-MT* shows a synergistic effect, as seen in transgenic soybean seeds where the α-tocopherol content increased 8-fold, with α-tocopherol comprising over 95% of total tocopherols [95]. This additive effect underscores the potential of combinatorial gene overexpression strategies to optimize vitamin E composition in crops. Additionally, the co-overexpression of *HPT* with *γ-TMT* not only boosts the total tocopherol content but also significantly increases the α-tocopherol proportion [75,96]. This suggests that the simultaneous upregulation of these genes effectively enhances both tocopherol quantity and quality, presenting a promising biofortification approach to increase the α-tocopherol content for improved nutritional outcomes. In summary, the overexpression of γ-TMT drives the robust conversion of γ-tocopherol to α-tocopherol in both vegetative and reproductive tissues, though the extent of this conversion can vary based on tissue-specific factors. Increasing α-tocopherol does not appear to diminish the levels or function of other antioxidant compounds, suggesting minimal competition among different antioxidant pathways. Moreover, a higher α-tocopherol content can enhance the plant’s resilience to biotic and abiotic stresses, potentially by boosting overall antioxidant capacity and reinforcing defense mechanisms. Current biofortification efforts for vitamin E primarily rely on conventional transformation techniques to overexpress key genes such as HPT and HGGT. While CRISPR/Cas9 gene editing holds great promise for precisely enhancing these pathways, its large-scale application is presently limited by the lack of identified negative regulatory factors in the vitamin E biosynthetic pathway. Once these factors are discovered, CRISPR-based approaches could be deployed to inactivate them, offering a more targeted and potentially scalable strategy for boosting the vitamin E content in staple crops.

Synthesizing the findings of various research reports, it becomes evident that the outcomes of biofortification using different strategies vary significantly (Table 1). Notably, the enhancement effects of HGGT and γ-TMT stand out. This variability suggests the presence of complex regulatory mechanisms within plants [97]. Consequently, these observations have encouraged researchers to pursue more detailed investigations into the genetic regulation of vitamin E biosynthesis.

## 5. Perspectives

Vitamin E is predominantly synthesized within the plastids, yet certain genes associated with its synthesis pathway are also located in other cellular compartments, such as the cytoplasm. The synthesis process necessitates the translocation of some substrates or products across the plastid membrane to the endoplasmic reticulum or other organelles for functional roles [100]. Despite the importance of this process, there is a notable scarcity of reports on proteins or transporters in plants that bind or transport vitamin E [101]. To date, the SlTBP protein found in tomato represents the only identified plant protein involved in the intracellular transport of α-tocopherol. This plastidial protein also plays a role in regulating lipid transport within tomato and is expressed in source leaves, sink leaves, and fruits. Nevertheless, the precise mechanisms by which SlTBP contributes to vitamin E transport require further elucidation [102]. The transport of precursor substances crucial for vitamin E synthesis is a significant focus for future research. HGA originates from the breakdown of Tyr within the shikimate pathway, where Tyr is transformed into HPP by TAT, followed by the conversion of HPP into HGA by HPPD. Recent findings indicate that TAT1 is localized in the cytoplasm [103], and HPPD has been identified in both the chloroplast and cytoplasm [103,104], suggesting that HGA synthesis may occur entirely or partially in the cytoplasm before being transported to the plastid for vitamin E synthesis via an as-yet unidentified transporter. Furthermore, the identification of HGO in soybean within the cytoplasm suggests that, at least in soybeans, a mechanism exists for transporting HGA from the cytosol to the plastid for vitamin E synthesis [60].

PDP is derived from chlorophyll synthesis and degradation pathways, yet there is limited research on the relationship between CHLSYN, CHLase, and vitamin E synthesis. Additionally, no studies confirm a direct role of chlorophyll degradation in vitamin E synthesis. Thus, producing significant quantities of PDP through chlorophyll degradation to boost vitamin E production presents a key challenge for future genetic engineering in vitamin E biosynthesis.

In conclusion, future research on vitamin E biosynthesis should prioritize the following: (1) identifying new genes involved in vitamin E synthesis to deepen our understanding of the biosynthetic pathway; (2) integrating biofortification strategies with the genetic framework of vitamin E-related genes, with a focus on regulatory mechanisms; and (3) developing novel tools or methodologies to study the vitamin E transporters and precursor transport, which will advance our knowledge and facilitate further progress in vitamin E biofortification.

## Figures and Tables

**Table 1:** Summary of vitamin E biofortification.

**Genes Involved in Biofortification**	**Promoters Type**	**Target Plant**	**Biofortification Effect**	**References**
*Arabidopsis HPPD*	constitutive CaMV 35S	Arabidopsis	Tocopherol in leaves increased to 1.15~1.37-fold. Tocopherol in seeds increased to 1.10~1.11-fold.	[59]
*Arabidopsis HPPD*	seed-specific pDC3		Tocopherol in seeds increased to 1.24~1.28-fold.	
*E. coli TyrA*	constitutive CaMV 35S		Tocopherol in leaves increased to 2~3-fold.	[98]
*Arabidopsis HPPD + E. coli TyrA*			Tocopherol in leaves increased to 1.5-fold.	
*Hordeum vulgare HPPD*		Tobacco	Tocopherol in leaves increased to 2-fold.	[61]
*Arabidopsis HPPD*	seed-specific napin	Arabidopsis	Tocopherol in seeds increased to 1.09-fold.	[58]
*E. coli TyrA*			Tocopherol in seeds increased to 1.2-fold.	
*Arabidopsis VTE2*			Tocopherol in seeds increased to 1.41-fold.	
*Arabidopsis HPPD + Arabidopsis VTE2*			Tocopherol in seeds increased to 1.46-fold.	
*Arabidopsis HPPD + E. coli TyrA*			Tocopherol in seeds increased to 1.8-fold.	
*E. coli TyrA + Arabidopsis VTE2*			Tocopherol in seeds increased to 1.8-fold.	
*Arabidopsis HPPD + E. coli TyrA + Arabidopsis VTE2*			Vitamin E in seeds increased to 2.88-fold.	
*E. coli TyrA*		Canola	Tocopherol in seeds increased to 1.6-fold.	
*Arabidopsis HPPD + E. coli TyrA*			Vitamin E in seeds increased to 2.4-fold.	
*Arabidopsis HPPD + E. coli TyrA + Glycine max. VTE2*			Vitamin E in seeds increased to 2-fold.	
*E. coli TyrA*		Soybean	Tocopherol in seeds increased to 1.1-fold.	
*Arabidopsis VTE2*			Tocopherol in seeds increased to 1.16-fold.	
*Arabidopsis HPPD + E. coli TyrA*			Vitamin E in seeds increased to 2.6-fold.	
*Arabidopsis HPPD + E. coli TyrA + Glycine max. VTE2*			Vitamin E in seeds increased to 3.3-fold.	
*Arabidopsis HPPD + E. coli TyrA + Arabidopsis VTE2*			Vitamin E in seeds increased to 3-fold.	
*Arabidopsis DXS*	constitutive CaMV 35S	Arabidopsis	Vitamin E in seeds increased to 2-fold.	[66]
*Arabidopsis DXS*			Vitamin E in leaves of mature plants showed no significant increase.	[67]
*Arabidopsis DXR*			Vitamin E showed no significant change.	[68]
*Zea mays PORB2*	Ubi	Maize	Tocopherol in leaves and seeds increased by 1.28~1.50 and 1.18~1.19-fold.	[50]
*Arabidopsis VTE7*	constitutive CaMV 35S	Arabidopsis *Atvte7*	Tocopherol in leaves increased to 3.6-fold.	[36]
*Zea mays VTE7*			Tocopherol in leaves increased to 6.9-fold.	
*Arabidopsis VTE5*	seed-specific napin	Arabidopsis *Atvte5*	Vitamin E in seeds increased to 3.2-fold.	[70]
*Arabidopsis VTE6*	constitutive CaMV 35S	Arabidopsis	Vitamin E in seeds increased to 1.15-fold.	[47]
*Arabidopsis VTE2*			Tocopherol in leaves increased to 3~4.4-fold.	[72]
*Arabidopsis VTE2 + Arabidopsis VTE4*			Tocopherol in leaves increased by 1.8~2.1-fold.	
*Arabidopsis VTE7 + Arabidopsis VTE2*			Tocopherol in leaves increased by 5.9~6.4-fold.	[36]
*Arabidopsis VTE2*		Tobacco	α-Tocopherol in leaves increased by 5.4-fold.	[71]
*Arabidopsis VTE1*			α-Tocopherol in leaves increased by 4-fold.	
*Arabidopsis VTE1 + Arabidopsis VTE2*			α-Tocopherol in leaves increased by 7.1-fold.	
*Arabidopsis VTE1*		Lettuce	Tocopherol in leaves increased to 2~2.7-fold.	[73]
*Arabidopsis VTE2*			Tocopherol in leaves increased to 2-fold.	
*Arabidopsis VTE1*		Indica rice ASD16	Tocopherol in leaves increased to 2.2~3.26-fold.	[74]
*Arabidopsis VTE2*			Tocopherol in leaves increased to 2.8~3.5-fold.	
*Arabidopsis VTE1 + Arabidopsis VTE2*			Tocopherol in leaves increased to 4.3~5.3-fold.	
*Arabidopsis VTE2 + Arabidopsis VTE4*		Potato	α-Tocopherol content in tubers increased to 2.6~2.8-fold, while that in leaves rose to 4.8~5.1-fold.	[75]
*Lactuca sativa* L. *VTE2*		Arabidopsis	α-Tocopherol in leaves increased by 18-fold.	[76]
*Malus domestica Borkh. VTE2*		Tomato	α-Tocopherol in leaves and fruits increased to 3.6 and 1.7-fold.	[77]
*Hordeum vulgare* L. *HGGT*	endogenous D-Hordein	Barley	Tocotrienol in seeds increased to 1.10~1.15-fold.	[78]
*Hordeum vulgare* L. *HGGT*	constitutive CaMV 35S	Arabidopsis	Vitamin E in leaves increased to 10~15-fold.	[79]
*Hordeum vulgare* L. *HGGT*	strong embryo-specific	Maize	Tocotrienol in seeds increased to 6-fold.	
*Hordeum vulgare* L. *HGGT*	strong seed specific	Soybean	Tocotrienol in seeds increased to 8~10-fold.	[80]
*Hordeum vulgare* L. *HGGT*	seed-specific napin	Cotton	Cottonseed vitamin E content exhibited a 2~3-fold enhancement.	[7]
*Hordeum vulgare* L. *HGGT + Glycine max VTE4*	strong seed specific	Soybean	Tocotrienol in seeds increased to 7-fold.	[80]
*Hordeum vulgare* L. *HGGT*	seed-specific napin	Canola	Vitamin E in seeds increased to 4.19-fold.	[99]
*Oryza sativa* L. *HGGT*	constitutive CaMV 35S	Tobacco	Vitamin E in leaves increased to 3.4~4.7-fold.	[81]
*Hordeum vulgare* L. *HGGT*	——	Maize	Tocotrienol in kernels and crude oil increased by 18-fold.	[82]
*Arabidopsis VTE4*	seed-specific carrot DC3	Arabidopsis	α-Tocopherol in seeds increased by more than 80-fold.	[83]
*Arabidopsis VTE4*	constitutive CaMV 35S	Lettuce	α-/γ-Tocopherol ratio in T0 plants reached a maximum of 320.	[84]
*Arabidopsis VTE4*	16s rRNA	Tobacco chloroplasts	α-Tocopherol in seeds increased to 9.6-fold.	[85]
*Arabidopsis VTE4*	constitutive CaMV 35S	*Brassica juncea*	α-Tocopherol in seeds increased by more than 6-fold.	[86]
*Brassica napus VTE4*	seed-specific	Soybean	α- and β-Tocopherol in T2 seeds increased to 11.1 and 18.9-fold.	[87]
*Arabidopsis VTE4*	constitutive CaMV 35S		α-Tocopherol in seeds increased to 4-fold.	[88]
*Perilla frutescens VTE4*	seed-specific		α-Tocopherol in T2 seeds increased to 10.4-fold.	[89]
*Medicago sativa VTE4*	constitutive CaMV 35S	*Medicago sativa*	α-Tocotrienol in leaves increased by 0.6~2.4-fold.	[90]
*Medicago sativa VTE4*		Arabidopsis	α-Tocopherol in seeds was at least 10-fold.	
*Medicago sativa VTE4*		*Medicago sativa*	α-Tocopherol and total tocopherol in leaves increased to 1.36 and 1.31-fold.	[91]
*Glycine max. VTE4*	Ubi	Maize	α-Tocopherol in seeds increased by 3~4.5-fold.	[100]
*Glycine max. VTE4*		Arabidopsis	α-Tocopherol in seeds increased by 4~6-fold.	
*Zea mays VTE4*	embryo-preferred Glb1	Maize	α-Tocopherol in seeds increased by 6.5-fold.	[92]
*Zea mays VTE4*		Arabidopsis	α-Tocopherol in seeds increased by 4~5-fold.	
*Arabidopsis VTE3*	seed-specific	Soybean	γ-Tocopherol increased from 50% to 80%.	[95]
*Arabidopsis VTE3 + Arabidopsis VTE4*			α-Tocopherol increased by more than 8-fold.	
